# Clinical outcomes and survival in patients with NSCLC and EGFR exon 20 mutations: evidence from real-world clinical practice in a retrospective study in Galicia

**DOI:** 10.3389/fonc.2026.1677766

**Published:** 2026-02-23

**Authors:** Begoña Campos Balea, Martín Emilio Lázaro Quintela, Ma Carmen Areses Manrique, Cristina Azpitarte Raposeiras, Jorge García-González, Francisco Javier Afonso Afonso, Manuel Fernández-Bruno, Natalia Fernández Núñez, Raquel Romero Van der Schoot, Lucía Santomé Couto

**Affiliations:** 1 Lucus Augusti University Hospital, Lugo, Spain; 2 Vigo University Hospital Complex, Vigo, Spain; 3 University Clinical Hospital of Ourense, Ourense, Spain; 4 Pontevedra University Hospital Complex, Pontevedra, Spain; 5 University Hospital Complex, Santiago de Compostela, Spain; 6 Translational Medical Oncology Group (ONCOMET), Health Research Institute of Santiago de Compostela (IDIS), Santiago de Compostela, Spain; 7 Biomedical Research Center in Cancer Network (CIBERONC), Carlos III Health Institute, Madrid, Spain; 8 University Hospital Complex, Ferrol, Spain; 9 University Hospital Complex of A Coruña, A Coruña, Spain; 10 Povisa Ribera Hospital, Vigo, Spain

**Keywords:** EGFR gene, exon 20, non-small cell lung cancer (NSCLC), prognosis, progression-free survival, treatment

## Abstract

**Background:**

Non-small cell lung cancer (NSCLC) with mutations in epidermal growth factor receptor exon 20 (EGFR Ex20Ins) is rare and resistant to conventional tyrosine kinase inhibitors (TKIs), limiting treatment options. This retrospective study in Galicia (Spain) evaluated the clinical characteristics, treatment response, and survival of patients with NSCLC and EGFR Ex20Ins.

**Methods:**

Data from patients diagnosed between 2013 and 2023, confirmed by next-generation sequencing (NGS) or polymerase chain reaction (PCR), were included. Overall survival (OS), progression-free survival (PFS), and the incidence of serious adverse events were assessed.

**Results:**

Of the 43 patients in the present study, 39 had metastatic disease and were considered for survival analysis. Among patients with metastatic disease, the median OS was 14.6 months (95% CI: 7–20 months), and the median PFS was 7.4 months (95% CI: 3–12 months) with first-line therapy (1L). Patients treated with platinum-based chemotherapy in the 1L achieved a median PFS of 9.8 months (95% CI: 3–27 months), whereas those receiving TKIs achieved a median PFS of 3.4 months (95% CI: 1–12 months). No significant differences in treatment response were found according to age or sex.

**Conclusions:**

The results show the limited effectiveness of TKIs in patients with EGFR Ex20Ins and highlight the need for specific therapies. Platinum-based chemotherapy performed better in preventing disease relapse.

## Introduction

Lung cancer is one of the leading causes of mortality worldwide (1). This disease can be classified into small cell lung cancer (SCLC) and non-small cell lung cancer (NSCLC), the latter accounting for 85% of all cases of lung cancer. NSCLC includes various histological subtypes, mainly adenocarcinoma, squamous cell carcinoma, and large-cell carcinoma. Despite advances in therapies and diagnostics, the five-year survival of patients with NSCLC remains less than 20% (2).

Epidermal growth factor receptor (EGFR), a transmembrane tyrosine kinase, is critical in the pathogenesis of NSCLC, as its activation triggers signaling pathways that promote cell proliferation, inhibit apoptosis, and favor angiogenesis (3). Activating mutations in EGFR, which are present in up to 10-15% of Western patients and 50% of Asian patients, generate constitutive signaling that drives tumor growth and survival (4). These mutations have prompted the development of specific tyrosine kinase inhibitors (TKIs), which improve relapse-free survival and quality of life in patients with NSCLC who present these genetic alterations (5).

EGFR exon 20 insertions (EGFR Ex20Ins) represent a specific and clinically significant subset of mutations in NSCLC, accounting for approximately 2-12% of all cases (5, 6). Early diagnosis of these mutations is essential to guide clinical management, as no specific clinical features allow the identification of patients with Ex20Ins and their differentiation from those with classical mutations (7, 8). Historically, molecular testing relied primarily on targeted polymerase chain reaction (PCR) assays, which were limited to detecting a predefined set of variants and therefore failed to identify a substantial proportion of Ex20Ins alterations. In contrast, next-generation sequencing (NGS) provides broader genomic coverage and detects a wider spectrum of Ex20Ins variants, significantly improving diagnostic accuracy and enabling more precise molecular characterization (9, 10).

Patients with EGFR Ex20Ins often have limited responses to TKIs due to intrinsic resistance in their mechanism of action, which restricts their therapeutic options (11, 12). Platinum-based chemotherapy has long been the standard of care, although with modest benefits in terms of survival and quality of life (8). Currently, the bispecific anti-EGFR/MET antibody, amivantamab, has become the preferred therapeutic option for these patients. Recent studies have explored TKIs, such as furmonertinib, zipalertinib, and sunvozertinib, which have shown promising efficacy against the resistance observed with conventional TKIs (13–15); however, clinical results in broad populations are still scarce (12, 16, 17).

Despite advances in understanding the molecular biology of NSCLC, publications on the clinical characteristics and outcomes of patients with EGFR Ex20Ins are limited due to their low frequency (18). The limited efficacy of first- and second-generation TKIs has excluded these patients from numerous clinical trials, complicating the development of phase III studies with large cohorts (19). Furthermore, detecting EGFR Ex20Ins is complex due to heterogeneity and the limitations of conventional sequencing methods (20).

In recent years, the implementation of NGS in clinical practice in Galicia (Spain) has improved the identification of patients with less frequent mutations, including EGFR Ex20Ins. This technological advance has significantly changed the diagnosis and therapeutic management of these rare mutations (10). The management of patients with EGFR Ex20Ins is constantly evolving, as reflected by shifting treatment patterns in recent years (3, 5, 21).

Therefore, it is essential to analyze the impact of the available diagnostic options and the treatments used to assess the expected benefit in patient survival outcomes. This real-life study aimed to identify the clinical characteristics, treatments received, response to different therapeutic lines, and survival of a cohort of Spanish patients enrolled in the Galician National System who have been diagnosed with EGFR Ex20Ins.

## Materials and methods

This study was reported in accordance with the ESMO-GROW guidelines (5) and received approval from the Committee on Ethics for Research with Medicines (CEIm) of the Autonomous Community of Galicia on June 30, 2023. A retrospective, analytical, observational study was conducted under routine clinical practice conditions. These patients were administered various therapeutic agents, and treatment effectiveness was evaluated in terms of survival and incidence of adverse effects.

The eligibility criteria for the study included a histologically confirmed diagnosis of NSCLC, a molecular diagnosis of EGFR Ex20Ins in the last 10 years, and the absence of neoplasia concomitant to lung cancer.

The source data were collected from Galician Health System (SERGAS) electronic medical records. Data collection was carried out between June 2023 and June 2024, including all patients who met the eligibility criteria between 2013 and 2023, using records from eight hospitals across the seven health areas of Galicia.

The primary endpoints of this study were obtained from patient medical records. Endpoints were applied at different stages of disease progression, including initial diagnosis and throughout successive lines of therapy (first [1L], second [2L], third [3L], and fourth [4L]). Endpoints were categorized into baseline characteristics, exposure, and clinical outcomes. Baseline characteristics included age, sex, smoking status, exposure to carcinogens (such as radon, smoke from wood stoves, tobacco smoke, industrial chemicals and other environmental or occupational agents with carcinogenic potential), family history, tumor histology, disease stage, and the presence of metastases. Tumor stage was extracted from the electronic medical records as documented by the treating oncologist at diagnosis. Staging in all participating centers followed the standard of care according to the TNM classification and was based on routinely performed imaging, including contrast-enhanced thoracic and abdominal Computed Tomography (CT), brain Magnetic Resonance Imaging (MRI) or CT, and Positron Emission Tomography-Computed Tomography (PET-CT) when clinically indicated.

Molecular diagnosis of EGFR Ex20Ins was performed according to routine clinical practice in the participating centers. The mutation was identified predominantly using PCR-based assays (Cobas^®^, Roche Diagnostics), which were employed in 93% of cases, while NGS platforms (Illumina^®^, Illumina Inc.) were used in approximately 7% of patients.

Exposure was defined based on the therapeutic agent administered to patients with metastatic disease at each stage. The endpoint outcomes assessed included disease progression, adverse events, overall survival (OS), and progression-free survival (PFS).

### Statistical analysis

Descriptive statistics were performed for all variables. Data were summarized using absolute and relative frequencies for qualitative variables and means, medians, and standard deviations for quantitative variables. OS analysis considered the time elapsed from the start of treatment, defined as surgery or radical radiotherapy in patients diagnosed with localized disease, and the start of systemic treatment in those with metastatic disease until death or loss to follow-up. PFS analyses were also performed, considering the start date of the corresponding line of therapy (1L, 2L, 3L, or 4L) until the time of disease progression or death of patients with metastatic disease who were candidates for receiving the lines of therapy described. These analyses were performed using the Kaplan-Meier (KM) method. The log-rank test was used for comparisons between groups. A Cox proportional hazards model was constructed to analyze the time to progression and overall survival and to investigate the relationships between predictor variables (age, sex, and smoking status) and time (in months) in patients who developed metastatic disease.

The chi-square test was used to assess the associations between the best response to treatment and sex. It was also used to assess the associations between adverse toxic events and sex. The relationship between the best response to treatment and age was evaluated with the ANOVA for independent samples. The relationships between toxic adverse events and age were also analyzed with Student’s t test.

No prior calculation of the sample size was performed since all available patients in the database who met the inclusion criteria were included. Given the study’s retrospective design, no specific statistical power was established.

All tests were performed with Stata^®^ 18.1 (Stata Corp, College Station, TX, USA), and GraphPad Prism^®^ 8 (GraphPad Software, San Diego, CA, USA) was used to generate some figures. A significance value of 95% was used, with p ≤ 0.05 considered statistically significant.

## Results

### Baseline characteristics

In this study, 43 patients with NSCLC were analyzed, 60% of whom were women, and the median age was 66.5 years (range: 44-88). A total of 58% had a history of smoking, 88% were diagnosed with adenocarcinoma, and 77% were in stage IV at diagnosis (**Table 1**). EGFR Ex20Ins were detected in 90.70% of the patients at initial diagnosis and in four patients during later phases. Diagnostic methods included PCR for 93% of the patients and NGS for 7 In 6 patients, a repeat biopsy was performed at the time of progression, occurring between 1 and 8 y after the initial diagnosis.

**Table 1:** Baseline characteristics of patients with NSCLC and EGFR exon 20 mutations.

Variables	Frequency (%)
Sex	
Male	17 (40)
Female	26 (60)
Tobacco use	
No	13 (30)
Yes	25 (58)
Unknown	5 (12)
Exposure to carcinogens	
No	25 (58)
Yes	5 (12)
Unknown	13 (30)
Family history	
No	33 (77)
Yes	6 (14)
Unknown	4 (9)
Tumor histology	
Adenocarcinoma	38 (88)
Squamous cell carcinoma	5 (12)
Tumor	
T1a	1 (2)
T1b	2 (5)
T1c	2 (5)
T2a	5 (12)
T2b	2 (5)
T3	7 (16)
T4	14 (33)
Tx	10 (23)
Adenopathy	
N0	5 (12)
N1	7 (16)
N2	9 (21)
N3	11 (26)
Nx	11 (26)
Metastasis	
M0	10 (23)
M1a	9 (21)
M1b	2 (5)
M1c	22 (51)
TNM system	
IB	1 (2)
IIB	2 (5)
IIIA	5 (12)
IIIB	1 (2)
IIIC	1 (2)
IVA	10 (24)
IVB	23 (53)
Total	43 (100)

### Exposure

This section describes the real-world treatment patterns and patient distribution across successive lines of therapy in the study cohort. Among the 43 patients included in the study, ten presented with localized disease at the time of diagnosis, eight of whom received local treatment with curative intent (surgery or radiotherapy). Of these patients initially diagnoses with localized disease, and six later developed metastatic relapse, resulting in a total of 39 patients (91%) with metastatic disease.

Despite presenting with metastatic disease, eight patients were unable to initiate antineoplastic therapy due to severe clinical deterioration (ECOG >2). Thus, only 31 patients were included in the 1L, 17 patients were included in the 2L, nine progressed to the 3L, and four received treatment in the 4L. The patient flow across lines of therapy is shown in **Supplementary Data 1**.

In the 1L, the platinum doublet combination was the predominant regimen, administered to 36% ofpatients. This treatment was frequently followed by the administration of afatinib, osimertinib, andimmunotherapy. In the 2L, platinum doublet and immunotherapy remained the most common therapeutic agents(24% and 18%, respectively). In the 3L, a total of 9 patients received treatment. The distribution of treatments was balanced, with afatinib, platinum doublet, and immunotherapy administered to 22% of patients. In the 4L, platinum doublet and immunotherapy were again the most commonly used therapeutic agents, administered to 75% of patients (**Supplementary Data 2**).

### Therapeutic results

The evaluation of the response to the different lines of therapy revealed that, as the disease progressed to more advanced stages, treatment efficacy decreased, and the amount of missing data increased, which would allow an accurate assessment of the therapeutic results. No significant associations were found between treatment efficacy and the sex or age of patients, although only men reached the 4L. Disease progression, including local progression, distant dissemination, and CNS involvement, varied by line of therapy (**Table 2**, **Supplementary Data 3**).

**Table 2:** Response to treatment by line of therapy, with analysis of sex and age in patients with NSCLC and EGFR exon 20 mutations.

Response to treatment	1L			2L			3L			4L		
	F (%)	Sex (p value)	Age (p value)	F (%)	Sex (p value)	Age (p value)	F (%)	Sex (p value)	Age (p value)	F (%)	Sex (p value)	Age (p value)
DP	11 (35)	0.916	0.644	6 (35)	0.506	0.164	5 (56)	0.495	0.976	1 (25)	NA	0.948
SD	9 (29)			6 (35)			2 (22)			2 (50)		
PR	11 (35)			3 (18)						1 (25)		
ND	0 (0)			2 (12)			2 (22)					
Total	31 (100)			17 (100)			9 (100)			4 (100)		

1L, first-line therapy; 2L, second-line therapy; 3L, third-line therapy; 4L, fourth-line therapy; DP, disease progression; F, frequency; NA, not applicable (statistical analysis not possible due to limited data); ND, no data; PR, partial response; SD, stable disease.

Sex was analyzed using ANOVA, and age was analyzed using Student’s t test. A p value of <0.05 was considered to indicate statistical significance.

Toxicity assessment revealed a low incidence of grade 3 or higher adverse events in all lines of therapy. In the 1L, 68% of patients (n=31) did not experience severe toxicity, whereas 32% (10 patients) developed grade 3–4 events. In the 2L, 82% of patients did not present severe toxicity, while 18% (3 patients) experienced grade 3–4 events. In the 3L, 88% of patients remained free of severe toxicity, and 13% (1 patient) developed grade 3–4 events. In the 4L, 75% of patients did not experience severe toxicity, while 25% (1 patient) presented grade 3–4 toxicity. No significant differences were detected between the first three lines of therapy in terms of sex (p=0.919, p=0.807, p=0.495, respectively) or between the first two lines of therapy in terms of age (p=0.128, p=0.901, respectively). In the 4L, the small sample size prevented the evaluation of the relationships with sex or age (**Table 3**).

**Table 3:** Frequencies of adverse effects (grade ≥3) by line of therapy and therapeutic agent in patients with NSCLC and EGFR exon 20 mutations.

Type of treatment	1L		2L		3L		4L	
	Yes	No	Yes	No	Yes	No	Yes	No
CHT	3 (10)	9 (29)	0 (0)	9 (53)	0 (0)	3 (33)	0 (0)	3 (75)
IO	1 (3)	2 (6)	1 (6)	2 (12)	0 (0)	2 (22)	NA	NA
CHT+IO	1 (3)	1 (3)	NA	NA	NA	NA	NA	NA
TKIs	2 (6)	8 (26)	1 (6)	3 (18)	1 (11)	2 (22)		
CT	3 (10)	1 (3)	1 (6)	0 (0)	NA	NA	1 (25)	0 (0)
Total	31		17		9		4	

1L, first-line therapy; 2L, second-line therapy; 3L, third-line therapy; 4L, fourth-line therapy; CHT, chemotherapy; CHT+IO, chemotherapy and immunotherapy; CT, clinical trial; TKIs, tyrosine kinase inhibitors. The percentages are relative to the number of patients per line of therapy.

### Overall survival

**Figure 1:**
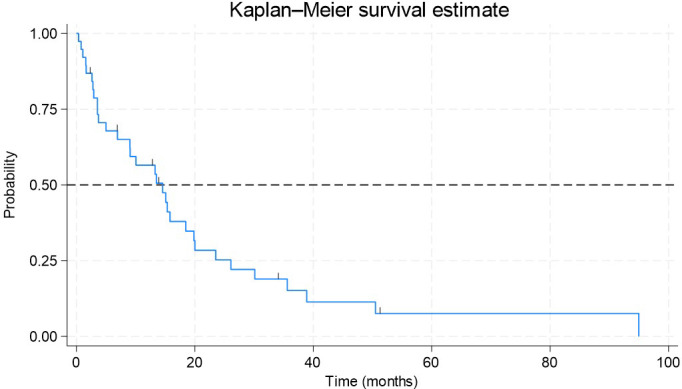
Kaplan–Meier estimate of overall survival (OS) in patients with metastatic NSCLC and EGFR exon 20 mutations. It shows probability on the vertical axis and time in months on the horizontal axis. The median OS in this population was estimated to be 14.6 months, with a 95% confidence interval (95% CI: 7-20 months).

A total of 39 patients with metastatic disease were included in the OS analysis. The median follow-up time at the end of the study was 17 months, with the duration from inclusion to study exit ranging from 0.4 to 95.0 months. During the follow-up period, 32 events were recorded, while six patients were censored. The median OS in this population was estimated to be 14.6 months, with a 95% confidence interval (95% CI: 7–20 months) (**Figure 1**).

The analysis using the Cox proportional hazards model, which was performed on the 38 patients with metastatic disease, did not reveal statistical significance in the relationships between the evaluated predictor variables and the time to death (p=0.530). Specifically, age, sex, and smoking status were not significantly associated with the probability of death from the time of diagnosis of metastatic disease.

### Progression-free survival

**Figure 2:**
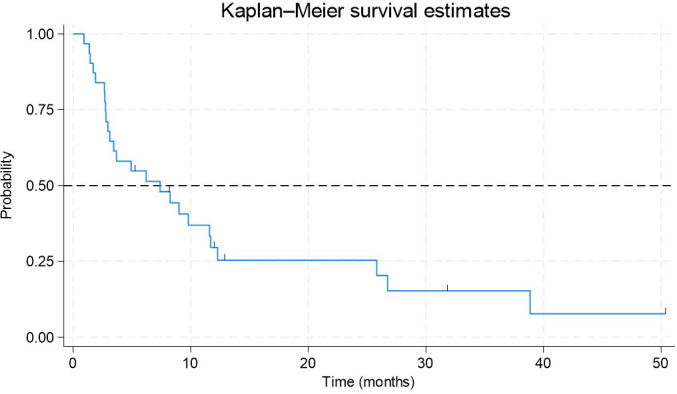
Kaplan–Meier estimate of progression-free survival in patients with metastatic NSCLC and EGFR exon 20 mutations receiving first-line therapy. It shows probability on the vertical axis and time in months on the horizontal axis. The median PFS in this population was estimated to be 7.4 months (95% CI: 3-12 months).

PFS analysis was performed on a total of 31 patients with metastatic disease who were treated with the 1L. The median follow-up time to the end of the study was 10.5 months, with the duration from inclusion to exit ranging between 0.9 and 50.3 months. During this period, 25 disease relapse events were recorded, while six patients were censored. The median PFS in this population was estimated to be 7.4 months (95% CI: 3–12 months) (**Figure 2**).

**Figure 3:**
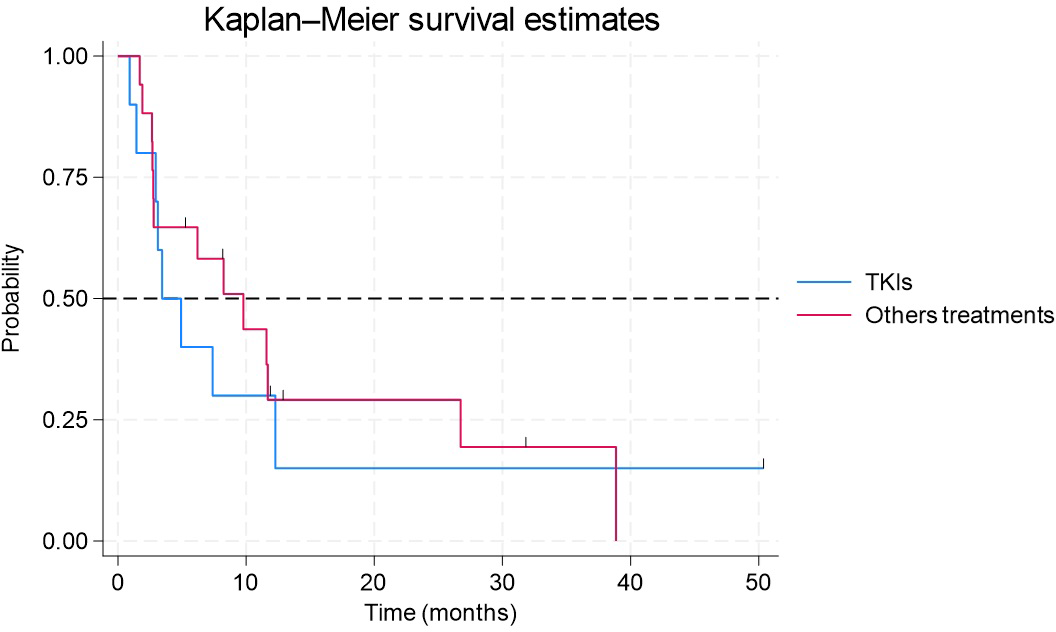
Kaplan–Meier estimate of progression-free survival in patients with metastatic NSCLC and EGFR exon 20 mutations receiving TKIs vs. other therapeutic agents in the 1L. For those treated with TKIs, the median follow-up was 9.9 months, with a median PFS of 3.4 months (95% CI: 1-12 months). Among patients in the other lines of therapy, the median follow-up was 10.9 months, and the median PFS was 9.8 months (95% CI: 3-27 months).

Ten patients received TKIs, and 17 were treated with other therapies in the 1L. For those treated with TKIs, the median follow-up was 9.9 months, with a median PFS of 3.4 months (95% CI: 1–12 months). Among patients in the other lines of therapy, the median follow-up was 10.9 months, and the median PFS was 9.8 months (95% CI: 3–27 months) (**Figure 3**). A log-rank test was performed to compare survival curves between patients treated with TKIs and those receiving other therapeutic agents in the 1L. No statistically significant differences in survival rates were observed between the evaluated groups (p = 0.745).

In the 2L, 17 patients with metastatic disease had a median follow-up of 7.9 months, with a median relapse-free survival of 3.4 months (95% CI: 1–6 months). In the 3L, nine patients were followed for a median of 3.9 months, with a median relapse-free survival of 2.5 months (95% CI: 0.5–5 months). In both lines of therapy, all patients experienced relapse without data censoring.

## Discussion

Our findings confirm that the currently available systemic therapies have limited effectiveness in patients with Ex20Ins, particularly in cases of metastatic disease. Although platinum-based chemotherapy produced a better treatment response compared to conventional TKIs, the overall benefit was modest and decreased progressively with each subsequent line of treatment. The survival outcomes observed in our patient group are consistent with the complex clinical course described in the literature for this specific molecular subgroup.

Our median OS of 14.6 months is consistent with previous studies reporting survival close to 16.2 months in patients with EGFR exon 20 mutations (18, 22). The PFS among patients receiving platinum-based chemotherapy in the 1L was 9.8 months, which agrees with the moderate efficacy reported in the literature (23). In contrast, conventional TKIs showed lower efficacy, with a median PFS of 3.4 months, reflecting the resistance of exon 20 mutations (22). These results highlight the need to administer more effective treatments to these patients, such as furmonertinib, zipalertinib, sunvozertinib, and amivantamab, which have shown clinical benefits (8, 13–15, 17).

In routine clinical practice, survival in patients with NSCLC who have classic EGFR mutations differs markedly from that observed in those with rare EGFR alterations other than Ex20Ins. A multicenter analysis of 468 patients with advanced NSCLC revealed that classic mutations (ex19del, L858R) were associated with a median OS of 22.7 months, considerably higher than the 12.0 months observed in patients with rare variants such as G719X, L861Q, or S768I (24). Similarly, in a cohort of patients treated with first-line osimertinib, median OS reached 39.1 months for ex19del and 30.1 months for L858R, while rare mutations only reached 15.6 months (25). These differences reflect intrinsic biological heterogeneity and varying sensitivity to TKIs (26).

In clinical trials, patients with EGFR Ex20Ins NSCLC assigned to control groups have also shown clearly unfavorable clinical outcomes. In the phase 3 PAPILLON trial, the chemotherapy-only arm (carboplatin/pemetrexed) achieved a median PFS of 6.7 months (27). Similarly, in external comparator analyses of the CHRYSALIS study, patients treated with standard regimens showed a median OS of 12.52 months in the European-American cohort (28) and 11.63 months in Chinese patients (29), along with very low objective response rates, ranging from 1.0% to 17.0% (28, 30). Taken together, these external parameters indicate that the median OS of 14.6 months observed in our cohort is within the lower range reported for rare EGFR mutations and closely aligns with the survival outcomes observed in the control groups of clinical trials.

The low incidence of serious adverse events in our study indicates a favorable toxicity profile, although even low toxicity levels may affect patients’ quality of life and adherence to treatment regimens. Recent studies highlight that emerging therapies such as furmonertinib, zipalertinib, sunvozertinib and amivantamab, in addition to being effective in specific populations, present manageable toxicity profiles, which optimize clinical outcomes and patient well-being (13–15, 17). The lack of variations in response according to age and sex suggests their safe applicability in various subpopulations, potentially expanding their clinical use.

Several studies have shown a higher prevalence of NSCLC among men (31). However, in our cohort of patients, this trend was reversed, with females representing 60.47% of patients. This finding is particularly notable, except in the 4L, which included only men.

A strength of our study is its basis in real-world data from patients with NSCLC in clinically treated in Galicia, providing an authentic perspective on the management of EGFR Ex20Ins. Real-world studies are essential for understanding the effectiveness of treatments in diverse populations and reflecting daily clinical complexities (7, 18, 28, 32, 33). Our findings offer practical information for other centers treating patients with NSCLC and Ex20Ins, improving the understanding of the efficacy and tolerability of different therapeutic agents among these patients. This is relevant given the low frequency of these mutations and the underrepresentation of affected patients in clinical trials and may improve clinical decision-making (34).

Our study has several limitations, including a small sample size, which restricts its generalizability and may affect its statistical power (35). In addition, the retrospective design introduces potential selection bias and inconsistencies in data collection, affecting internal validity. The lack of data from patients in advanced lines of therapy also limits the complete assessment of therapeutic efficacy. Furthermore, mutation identification in our study was performed mainly by PCR, although the current standard is NGS, which offers a more exhaustive detection. Although rapid and sensitive, PCR may fail to detect more than 50% of EGFR Ex20Ins (36, 37). Furthermore, since this is a retrospective study based on data extracted from clinical records, assessing toxicities has certain inherent limitations. In the context of routine clinical practice, low-grade adverse events that do not affect therapeutic management are often underreported, making it difficult to fully and accurately characterize the toxicity profile of the therapeutic agents evaluated. Therefore, it is important to highlight the need to implement NGS more widely in clinical practice, ideally in all hospital centers. NGS allows a more exhaustive characterization of genomic alterations and a more sensitive and specific detection of EGFR Ex20Ins. Several studies have highlighted the importance of systematically integrating NGS into the routine diagnosis of lung cancer since its wide availability contributes to the early and accurate identification of genetic variants that could go unnoticed when using more limited molecular techniques (38). In this way, access to more effective targeted therapies would be improved, and personalized therapeutic strategies could also be optimized, positively impacting the survival and quality of life of patients with NSCLC and EGFR Ex20Ins.

In our study, only 6 of the 43 patients underwent rebiopsy after tumor progression, reflecting the low frequency with which this practice is implemented in the clinic. Rebiopsy is a key tool in the management of NSCLC, as it allows for the identification of tumor resistance mechanisms that may have arisen during treatment and the detection of rare mutations that might not have been initially identified due to limitations in the diagnostic methodology. This is especially relevant for EGFR Ex20Ins, as more exhaustive molecular analysis using techniques such as NGS could increase the detection of these alterations and expand the therapeutic options for patients.

Prospective studies are needed to allow for greater control of variables and detailed follow-up in patients with EGFR Ex20, providing solid evidence of the efficacy and safety of new therapies (39). Agents such as poziotinib, mobocertinib and amivantamab have shown potential in initial trials, improving response and survival rates, but further research is required to validate their efficacy in clinical practice (8, 17). Participation in trials and collaboration between centers are key to developing effective therapies and optimizing outcomes for these patients.

## Conclusions

This study revealed that patients with NSCLC and EGFR Ex20Ins have limited overall survival due to their poor prognosis and the lack of effective targeted therapies. No significant differences in survival were observed according to age, sex or response to treatment. Patients with EGFR exon 20 mutations need more effective therapies. Drugs such as amivantamab and emerging agents such as furmonertinib, zipalertinib and sunvozertinib show promising potential according to preliminary studies. It is necessary to optimize diagnosis to ensure access to specific treatments and improve clinical outcomes.

## Data Availability

The data supporting the findings of this study are available from the corresponding author upon request.

## Supplementary material

Supplementary Data 1 The flow chart of the study. 1L, first-line therapy; 2L, second-line therapy; 3L, third-line therapy; 4L, fourth-line therapy. Icons by Canva (Canva.com).

Supplementary Data 2 Frequency of use of therapeutic agents across different lines of therapy in patients with NSCLC and EGFR exon 20 mutations. 1L, first-line therapy; 2L, second-line therapy; 3L, third-line therapy; 4L, fourth-line therapy. CHT, chemotherapy; CHT+IO, chemotherapy and immunotherapy; CT, clinical trial; TKIs, tyrosine kinase inhibitors.

Supplementary Data 3 Patterns of disease progression by line of therapy in patients with NSCLC and EGFR exon 20 mutations. 1L, first-line therapy; 2L, second-line therapy; 3L, third-line therapy; 4L, fourth-line therapy; NA, not applicable.
