# Supplementary figures and images for: Clinical outcomes and survival in patients with NSCLC and EGFR exon 20 mutations: evidence from real-world clinical practice in a retrospective study in Galicia
Source: Front Oncol. 2026 Feb 23;16:1677766. doi: 10.3389/fonc.2026.1677766 (PMC12967965; doi:10.3389/fonc.2026.1677766)

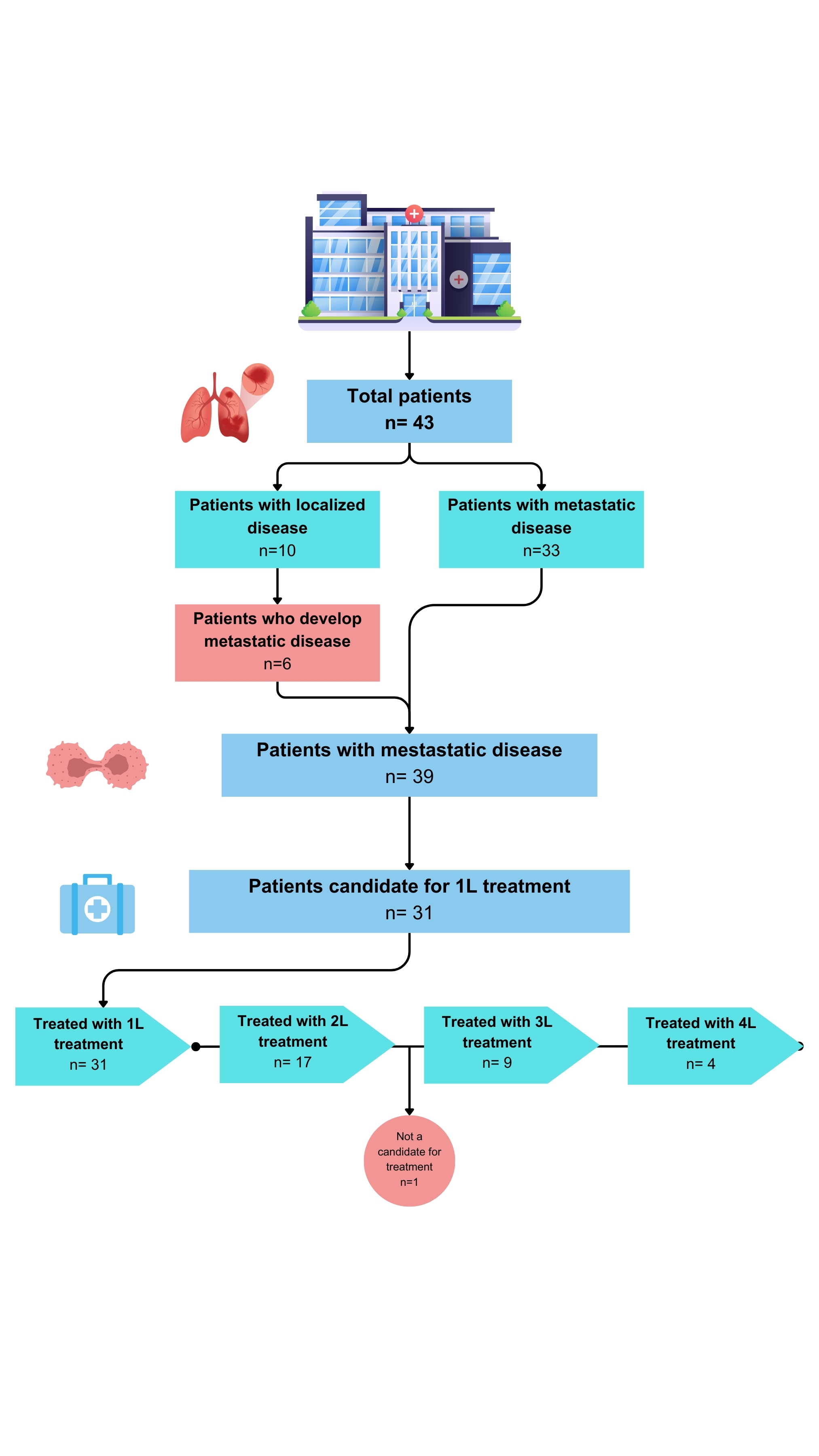

Supplement: Supplementary Data 1 — The flow chart of the study. 1L, first-line therapy; 2L, second-line therapy; 3L, third-line therapy; 4L, fourth-line therapy. Icons by Canva (Canva.com). [file SupplementaryFile1.jpeg]
