# Supplementary material for: Clinical outcomes and survival in patients with NSCLC and EGFR exon 20 mutations: evidence from real-world clinical practice in a retrospective study in Galicia
Source: Front Oncol. 2026 Feb 23;16:1677766. doi: 10.3389/fonc.2026.1677766 (PMC12967965; doi:10.3389/fonc.2026.1677766)
Supplement: Supplementary Data 2 — Frequency of use of therapeutic agents across different lines of therapy in patients with NSCLC and EGFR exon 20 mutations. 1L, first-line therapy; 2L, second-line therapy; 3L, third-line therapy; 4L, fourth-line therapy. CHT, chemotherapy; CHT+IO, chemotherapy and immunotherapy; CT, clinical trial; TKIs, tyrosine kinase inhibitors. [file SupplementaryFile2.docx]

**Supplementary Data 2.** Frequency of use of therapeutic agents across different lines of therapy in patients with NSCLC and EGFR exon 20 mutations

|  |  | **Frequency (%)** | | | |
| --- | --- | --- | --- | --- | --- |
| **Pharmacological group** | **Drug** | **1L** | **2L** | **3L** | **4L** |
| TKIs | Mobocertinib |  | 1 (5.88) | 1 (11.11) |  |
|  | Afatinib | 3 (9.68) |  | 2 (22.22) |  |
|  | Gefitinib | 2 (6.45) |  |  |  |
|  | Erlotinib | 2 (6.45) | 2 (11.76) |  |  |
|  | Osimertinib | 3 (9.68) | 1 (5.88) |  |  |
| CHT+IO | Platinum doublet and Immunotherapy | 2 (6.45) |  |  | 3 (75.00) |
| CT | Clinical trial | 4 (12.90) | 1 (5.88) |  | 1 (25.00) |
| IO | Immunotherapy | 3 (9.68) | 3 (17.65) | 2 (22.22) |  |
| CHT | Pemetrexed | 1 (3.23) |  | 1 (11.11) |  |
|  | Vinorelbine |  | 1 (5.88) |  |  |
|  | Taxol |  | 1 (5.88) |  |  |
|  | Taxotere |  | 1 (5.88) | 1 (11.11) |  |
|  | Taxotere and Nintedanib |  | 1 (5.88) |  |  |
|  | Gemcitabine |  | 1 (5.88) |  |  |
|  | Platinum doublet | 11 (35.48) | 4 (23.53) | 2 (22.22) |  |
|  | **Total** | 31 (100.00) | 17 (100.00) | 9 (100.00) | 4 (100.00) |

1L: first-line therapy; 2L: second-line therapy; 3L: third-line therapy; 4L: fourth-line therapy. CHT: chemotherapy; CHT+IO: chemotherapy and immunotherapy; CT: clinical trial; TKIs: tyrosine kinase inhibitors.
