# Supplementary material for: Clinical outcomes and survival in patients with NSCLC and EGFR exon 20 mutations: evidence from real-world clinical practice in a retrospective study in Galicia
Source: Front Oncol. 2026 Feb 23;16:1677766. doi: 10.3389/fonc.2026.1677766 (PMC12967965; doi:10.3389/fonc.2026.1677766)
Supplement: Supplementary Data 3 — Patterns of disease progression by line of therapy in patients with NSCLC and EGFR exon 20 mutations. 1L, first-line therapy; 2L, second-line therapy; 3L, third-line therapy; 4L, fourth-line therapy; NA, not applicable. [file SupplementaryFile3.docx]

**Supplementary Data 3.** Patterns of disease progression by line of therapy in patients with NSCLC and EGFR exon 20 mutations

|  | **1L** | **2L** | **3L** | **4L** |
| --- | --- | --- | --- | --- |
|  | **Frequency (%)** | | | |
| Progression |  |  |  |  |
| Local | 7 (22.58) | 2 (11.76) | 1 (11.11) | 1 (25.00) |
| Metastatic | 13 (41.94) | 10 (58.82) | 4 (44.44) | 1 (25.00) |
| CNS involvement | 1 (3.23) | NA | 1 (11.11) | 1 (25.00) |
| No data | 10 (32.26) | 5 (29.41) | 3 (33.33) | 1 (25.00) |
| **Total** | 31 (100.00) | 17 (100.00) | 9 (100.00) | 4 (100.00) |

1L: first-line therapy; 2L: second-line therapy; 3L: third-line therapy; 4L: fourth-line therapy; NA: not applicable
